# PepeSearch: Semantic Data for the Masses

**DOI:** 10.1371/journal.pone.0151573

**Published:** 2016-03-11

**Authors:** Guillermo Vega-Gorgojo, Martin Giese, Simen Heggestøyl, Ahmet Soylu, Arild Waaler

**Affiliations:** 1 Department of Informatics, University of Oslo, Oslo, Norway; 2 Faculty of Computer Science and Media Technology, Norwegian University of Science and Technology, Gjøvik, Norway; University of Amsterdam, NETHERLANDS

## Abstract

With the emergence of the Web of Data, there is a need of tools for searching and exploring the growing amount of semantic data. Unfortunately, such tools are scarce and typically require knowledge of SPARQL/RDF. We propose here PepeSearch, a portable tool for searching semantic datasets devised for mainstream users. PepeSearch offers a multi-class search form automatically constructed from a SPARQL endpoint. We have tested PepeSearch with 15 participants searching a Linked Open Data version of the Norwegian Register of Business Enterprises for non-trivial challenges. Retrieval performance was encouragingly high and usability ratings were also very positive, thus suggesting that PepeSearch is effective for searching semantic datasets by mainstream users. We also assessed its portability by configuring PepeSearch to query other SPARQL endpoints.

## Introduction

The last few years we have witnessed the emergence of the Web of Data [1]–a giant, global and interconnected data space spanning all sorts of topics, such as media, publications or geographic locations. Following the so-called Linked Data principles [2], publishers of any kind can contribute, with their datasets, to the Web of Data. Briefly, this process involves minting URIs for naming resources, preparing RDF [3] (Resource Description Framework) representations for lookup and linking to other related resources. Once a dataset is ready, a triple store is commonly employed for publishing Linked Data. Besides URI lookup, triple stores provide a SPARQL endpoint, a popular and convenient mechanism for querying a dataset using the SPARQL query language [4]. Although publishing Linked Data requires a non-trivial effort, it provides a generic and flexible mechanism for accessing, discovering and integrating data from different sources. As a result, there is an ongoing trend to publish Linked Data by private organizations and public bodies such as universities, broadcasters and governments [1].

With such a large volume of structured data available, it is possible to formulate semantic searches, i.e. referring to concepts instead of word occurrence as in text searches, for any imaginable topic. For example, we can ask *which Norwegian companies have ever had a net income of more than one billion Norwegian kroner*, *but no more than 4 employees* to the RDF version of the Norwegian Register of Business Enterprises (http://data.computas.com). Unfortunately, posing this query requires knowledge of SPARQL and of the underlying data structure, thus severely limiting the target audience. This is especially unfortunate in the case of Linked Open Government Data [5] since citizens have funded these initiatives and they should be able to take profit of them.

Given the effort invested in setting up a dataset in the Web of Data—assigning URIs, creating and reusing vocabularies, defining mappings and interlinking to other datasets—we should find new ways of facilitating the search of semantic data by mainstream users [6], i.e. novice to casual users that are computer literate and do not necessarily have knowledge of RDF or SPARQL. Unfortunately, most tools available are not appropriate for searchers having few-to-no technical skills [7]. Most are SPARQL query interfaces that require a user to learn how to write SPARQL syntax. We address this problem here with PepeSearch, a form-based tool for querying arbitrary SPARQL endpoints. PepeSearch is intended for mainstream users, so the user interface has been carefully designed to be highly usable, while at the same time supporting a considerable degree of expressivity. *Pepe* is the pet form of José, a common Spanish name; PepeSearch is thus a search tool for “the common man”.

The rest of the paper is organized as follows. In Section 2 we analyze the different approaches for querying semantic datasets. We depict the novel form-based interface of PepeSearch in Section 3. We present the results of a user study with PepeSearch and showcase its portability in Section 4. Finally, we include a discussion and plans for future work in Section 5.

## Related work

We can find different types of query specification interfaces for semantic datasets in the literature: SPARQL query editors, information retrieval-based interfaces, natural language interfaces and visual approaches. When analyzing the different alternatives, we are mainly interested in the usability and expressiveness criteria. Usability refers to learnable, efficient and intuitive user interfaces, while expressivity indicates the variety of queries that can be posed. Since there is a trade-off between both dimensions [8], some query expressivity is commonly sacrificed to achieve a usability gain. Besides, a search system for the Web of Data needs to be portable, i.e. no code changes should be required to be used in a new domain.

Table 1 summarizes the different alternatives found in the literature. A SPARQL editor is a simple solution that allows the use of the full expressivity of the query language. However, this is not an option in most cases since users have to know the SPARQL syntax. Further, querying a triple store needs familiarity with the underlying data schema. To overcome this limitation, context-aware editors like SparQLed [9] extract a structural summary of a dataset and then provide autocompletion and recommendations of structure elements such as class or property names—though knowledge of SPARQL is still required.

**Table 1:** Approaches for querying semantic datasets.

General approach	Approach	Usability	Expressivity	Examples	Comments
Formal query language	SPARQL editor	Very low	High	SparQLed [9]	Requires SPARQL knowledge
					Users are not aware of the data structure
					Simple solution
Information retrieval	Keyword-based search interfaces	High	Very low	SWSE [10]	Well-known approach
					Users are not aware of the data structure
Natural language processing	Question-answering systems	High	Natural language approximation	PowerAqua [11]	Ambiguities and linguistic variability
					Users are not aware of the data structure
					Complex solution
Visual	Graph-based query editors	Medium	High/Medium	NITELIGHT [13], QueryVOWL [14]	Natural choice for RDF data
					Not very usable for mainstream users
	Form-based query editors	High	Medium	Virtuoso facets [15], Explorator [16], tFacet [17], Rhizomer [18]	Difficult to translate SPARQL query patterns into a generic form interface
					Well-known approach

Information retrieval approaches mimic traditional keyword-based search, allowing users to pose bags-of-words as queries—hence expressivity is limited. The search system tries to match keywords to classes or instances, possibly exploiting relations in the dataset to create a structured SPARQL query—see SWSE [10] for an example. As an evolution, natural language approaches, e.g. PowerAqua [11], interpret the query as a whole with the aim of achieving the expressivity of natural language. However, ambiguities and linguistic variability limit its effectiveness [12], so existing approaches typically engage the user in feedback cycles or try to get approximate results. The challenges of information retrieval and natural language approaches for querying the Web of Data are thoroughly analyzed in [8].

Visual approaches comprise a wide range of user interfaces that allow the construction of SPARQL queries through the interaction with visual elements. The syntax of the language is hidden from the user, while available actions are limited so as to only produce valid queries. The majority of visual approaches can be classified either as graph-based or form-based. Graph-based query editors allow to visually construct a query by adding nodes (representing RDF classes or individuals) and arcs (representing RDF properties). There are many examples of graph-based interfaces, e.g. NITELIGHT [13] or QueryVOWL [14], since Linked Data can be represented as graphs. Nevertheless, there is evidence that mainstream users are not particularly comfortable with graph visualizations [19, 20], making this approach questionable for this user group from a usability point of view.

Finally, form-based interfaces allow the specification of queries through the use of text boxes, drop-down menus, radio buttons and other form elements. This approach is appealing to mainstream users, since they are commonly employed in everyday tasks such as flight booking. However, the challenge is the translation of SPARQL query patterns into a generic form interface. Preliminary attempts have reproduced faceted search [21] for semantic data by exploiting the vocabulary structure, e.g. Virtuoso Facets [15]. Unfortunately, expressivity is typically low, since queries can only include one concept. More recent developments have tried to overcome this limitation: Explorator [16] includes Boolean operators for combining facets; tFacet [17] supports so-called hierarchical facets by using additional menu forms to include facets from other classes; Rhizomer [18] provides pivoting operations, i.e. changing the focus of a query from one class to another. Overall, the effectiveness of these approaches is questionable for mainstream users, given their increased complexity and the scarcity of user studies to assess their benefits.

## PepeSearch: a portable form-based search interface for semantic datasets

We pursue to provide a highly usable search tool that actually hides the complexity of SPARQL/RDF, while ensuring syntactic correctness and making the user conscious of the data structure. Further, expressivity should be enough to cover a wide range of query types, although we do not aim to support all the SPARQL expressivity. In addition, the proposed search tool should be portable to an arbitrary dataset without requiring changes in the code.

### System architecture

PepeSearch is the tool we have devised to fulfill the aforementioned requirements. Its user interface corresponds to the category of form-based query editors (see Table 1). PepeSearch provides a multi-class search form that allows the searcher to set multiple constraints on any of the classes in the dataset.

**Fig 1:**
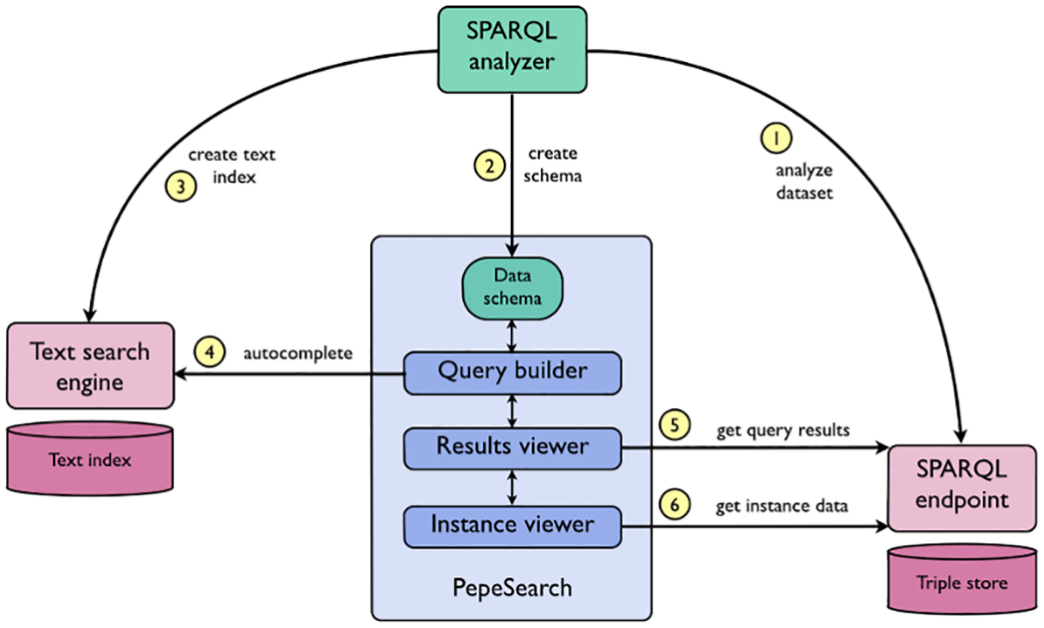
Logical architecture of Pepesearch.

The system architecture of PepeSearch is depicted in Fig 1. The SPARQL analyzer is employed in a bootstrapping stage to gather information about the dataset. A data schema in the standardized JavaScript Object Notation (JSON) format is generated from this process. This data schema is the basis for the creation of the multi-class form in the user interface component. For a given class, the data schema can be inspected to get a readable label, its datatype properties with appropriate ranges, and the connections to other classes through an object property or through a subclass relation. Furthermore, the SPARQL analyzer can optionally retrieve the string values of datatype properties which are then sent to a text search engine.

A system administrator will typically carry out this bootstrapping process and then set up a PepeSearch instance with the obtained data schema. There is no need to rerun the SPARQL analyzer unless there are unreflected structural changes in the RDF model of the triplestore, e.g. a new class is introduced, or new data that invalidates the obtained ranges of a numerical datatype. In any case, the data schema will still be usable and it is possible (and advisable) to schedule periodical updates based on the frequency of data changes.

**Fig 2:**
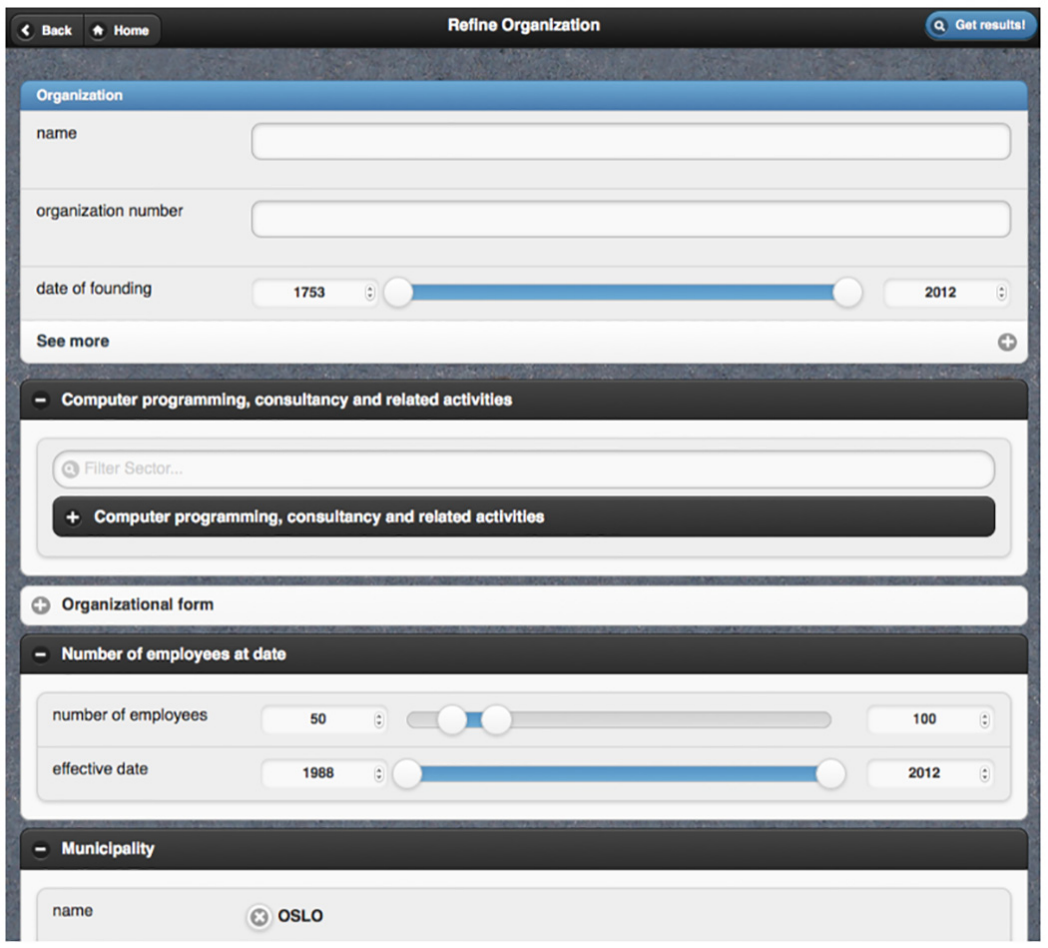
Snapshot of PepeSearch (query).

Once a PepeSearch instance is configured, the query builder relies on the obtained data schema to construct the view—see Fig 2. The user can restrict any of the fields of the presented classes in the form; appropriate ranges and term suggestions are exploited to reduce input errors. The text search engine is employed to make dynamic term suggestions during query specification—for example, after typing “os” in the municipality textbox, PepeSearch will suggest OS, OSLO, ØSTRE TOTEN, OSTERØY, and OSEN. In case of subclasses, the form includes controls for selecting a more general/specific concept—see the filter and the collapsible of “Computer programming, consultancy and related activities” in Fig 2.

**Fig 3:**
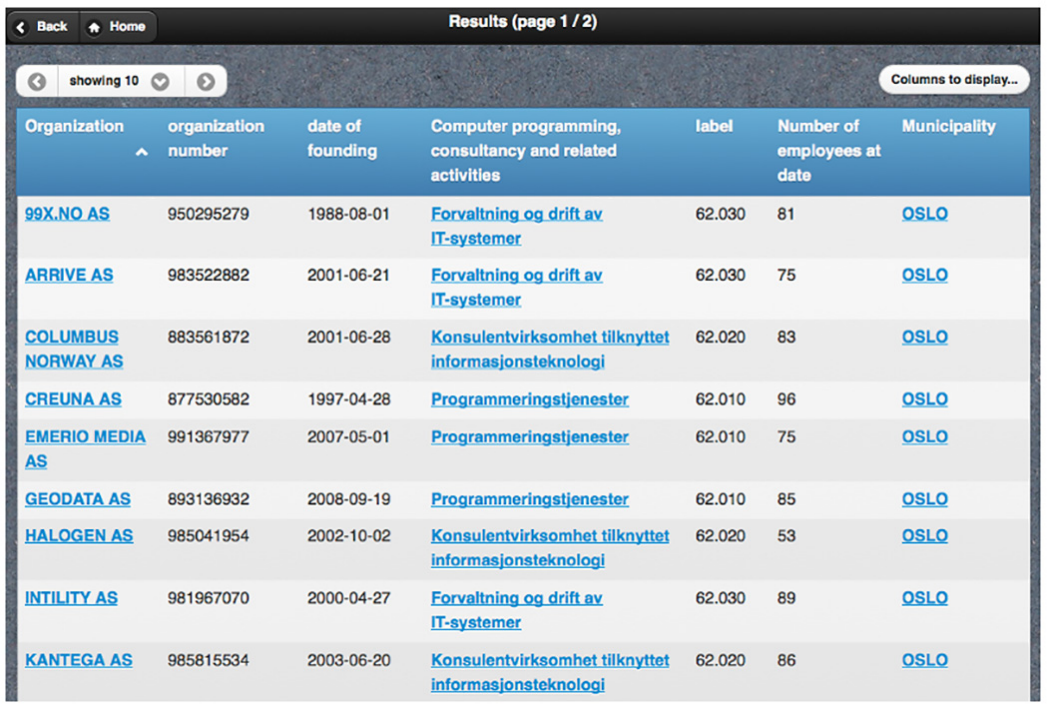
Snapshot of PepeSearch (results).

**Fig 4:**
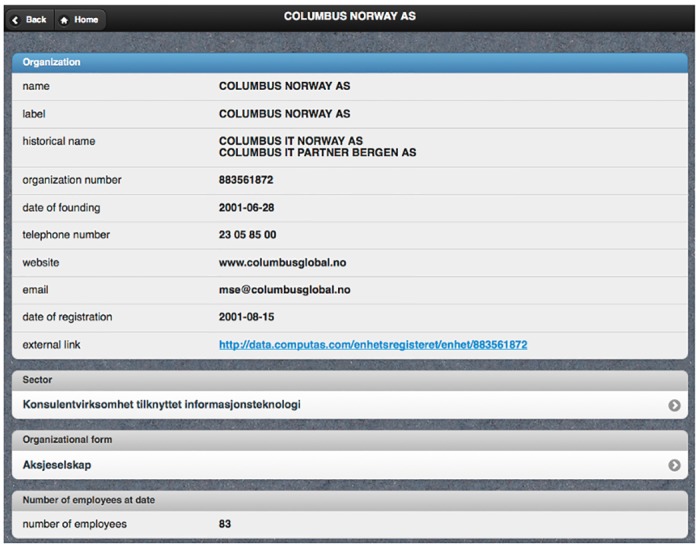
Snapshot of PepeSearch (instance).

After pushing the search button, the query builder generates a valid SPARQL conjunctive query containing all the user restrictions. The results viewer sends the query to the SPARQL endpoint and obtained results are then presented to the user in a tabular representation; this includes controls to paginate, add/remove columns and sort by any field—see Fig 3. Moreover, class instances are clickable, and the instance viewer is in charge of obtaining all the information available in the dataset and creating a corresponding view—see Fig 4.

### User interface design

**Fig 5:**
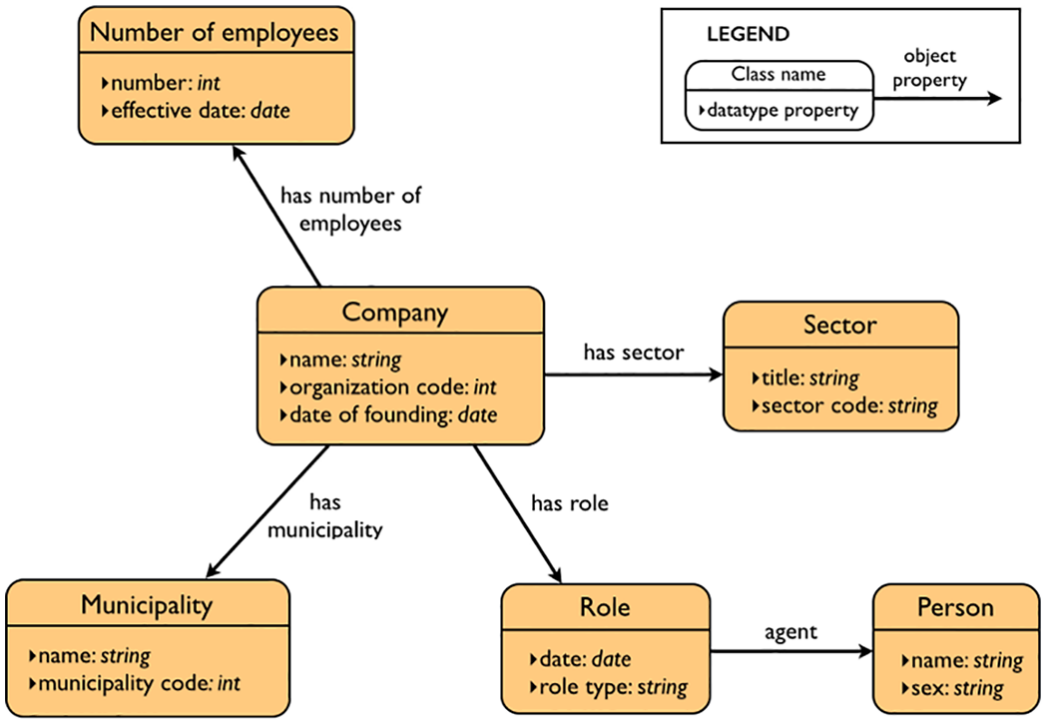
Vocabulary excerpt of the Norwegian Register of Business Enterprises.

For an arbitrary RDF class, PepeSearch creates a form block in which datatype properties are mapped to widget elements, e.g. text boxes for string literals or slide ranges for integers. In this way, a searcher can easily set restrictions on a class by manipulating the visual elements of the block form. Beyond refining a single class, further expressivity is required in many cases. In PepeSearch we address this challenge by providing new form blocks for each RDF class that is connected with an object property to the selected RDF class: for the vocabulary in Fig 5, PepeSearch will create a block form for the concept “Role'' when selected, as well as additional block forms for “Company'' and “Person''. Since form-based interfaces can become bloated with many form elements, we use collapsibles for these additional block forms—see Fig 2. The corresponding collapsibles can be expanded, thus allowing the searcher to set restrictions to the class attributes. Moreover, we provide controls for selecting a class type in the case of concept taxonomies. For instance, Fig 2 shows the “Computer programming, consultancy and related activities'' form block, one of the 380 subtypes of “Sector'' in the test vocabulary.

We illustrate our approach for the user interface with the following example: we want to find *which information technology consultancy companies in Oslo have between 50 and 100 employees*? The SPARQL analyzer has previously extracted the data structure from the Norwegian Register of Business Enterprises triple store—an excerpt of the vocabulary is shown in Fig 5. After that, the interaction with the system can be the following:

1. PepeSearch presents a list of the available classes.
2. The user selects “Company”.
3. PepeSearch presents a form composed of different classes: one for “Company” and another one for each directly connected concept in Fig 5. Each class includes controls for refining its attributes.
4. The user sets the following restrictions: “Computer programming, consultancy and related activities” in the sector hierarchy, “Oslo” in municipality, and “50–100 range” in the number of employees. Fig 2 shows a snapshot of this query.
5. The user pushes the search button.
6. PepeSearch generates a SPARQL query from the user input and sends it to the SPARQL endpoint.
7. With the response, PepeSearch prepares a suitable representation of the results—see Fig 3.
8. The user can navigate through the results by following the links, e.g. Fig 4 shows the information of one of the companies found.

### The SPARQL analyzer

The SPARQL analyzer is in charge of creating the data schema that will be employed to create the multi-class search form. Remarkably, this process is generally applicable to an arbitrary triplestore due to the self-describing nature of RDF, the schema-level queries in SPARQL, and to the generality of the mapping assumptions.

In a bootstrapping stage, the SPARQL analyzer submits a sequence of queries to the target triplestore in order to collect information about the dataset. First, a list of all the non-empty classes is obtained—this filtering is intended to limit the formulation of zero-results queries during user operation. Then, for each retrieved class the SPARQL analyzer get its subclass relations, object properties and datatype properties. In the case of numerical datatype properties, the SPARQL analyzer also collects the minimum and maximum values for each class in order to set appropriate slide ranges. Specifically, the main SPARQL queries employed for gathering that information are the following:

- Get the non-empty classes:

     SELECT DISTINCT ?class

     WHERE { ?instance a ?class . }

- Get the subclasses of a given class with URI class_uri

     SELECT DISTINCT ?subclass

     WHERE { ?subclass rdfs:subClassOf <class_uri> . }

- Get the distinct object properties and corresponding object classes for a given class with URI class_uri

     SELECT DISTINCT ?object_prop ?object_class

     WHERE { ?subject a <class_uri> .

        ?subject ?object_prop ?object .

        ?object a ?object_class . }

- Get the distinct datatype properties for a given class with URI class_uri

     SELECT DISTINCT ?literal_prop

     WHERE { ?subject a <class_uri> .

        ?subject ?literal_prop ?literal .

        FILTER (isLiteral(?literal))

### Implementation

The SPARQL analyzer is offered as a standalone component at https://github.com/simenheg/sparql-endpoint-analyzer. It analyzes the data structure of an arbitrary SPARQL endpoint and automatically generates the data schema and a term suggestions document. Although not required, some manual customization can be performed, especially to annotate secondary properties—for example, the company website is not available for search in Fig 2, but can be shown by pressing the “See more” button.

PepeSearch is coded in JavaScript and available at https://github.com/guiveg/pepesearch. We have employed the JQuery Mobile framework to simplify the development of the user interface, as well as to facilitate its deployment in most smartphone, tablet and desktop platforms. As for the text search engine, we have employed Apache Solr (http://lucene.apache.org/solr/), though this component is not required to run PepeSearch.

## PepeSearch in practice

### The Norwegian register of business enterprises

As part of the Semicolon II project, a collaboration of a number of public sector, industry, and academic partners in Norway, the Norwegian Register of Business Enterprises was published as Linked Open Data [22], (http://data.computas.com).

The triplestore contains administrative and accountancy data of more than 300K companies, amounting to almost 50M triples. The complexity of the schema is moderate/low, consisting of 11 classes, 16 object properties and 59 datatype properties (see an excerpt in Fig 1). We have set up a PepeSearch instance to query this triplestore, available at http://sws.ifi.uio.no/project/semicolon/search/.

In order to assess the effectiveness of PepeSearch, we arranged a search challenge with an award of a 1000 kroner book voucher for the best performer. This competition was advertised at the University of Oslo and at the Oslo Akershus University College. Eventually, 14 students and 1 postdoctoral researcher (6 female and 9 male) participated. 10 of them had a background in informatics and 5 in library studies. The average age was 25, ranging from 18 to 44. Participants sporadically made searches of Norwegian companies, although 2 of them reported higher frequency of searches. All of them were heavy users of computers and were very used to web searches. They had to register through a web questionnaire in order to participate in the study, and we sent them a link to five slides of PepeSearch a few days before the experiment.

The search challenge was carried out in a single session. We followed the ethics guidelines for conducting usability studies that are common in the field—see for instance [23]—and for this reason we did not seek approval of an institutional review board. As a result, participants in the study were informed that all their data would be anonymized and exclusively used for a research project on search interfaces. All of them gave their oral consent and carried out the scheduled search tasks. Participant consent was not recorded and we considered it was not necessary to do it, given that participants had previously registered in order to conduct the usability study. Indeed, they were volunteers and free to withdraw at any time.

We gave them some basic instructions for signing up and submitting the results of a search task. Each participant had to respond to three search tasks. These were randomly selected from the following pool:

- *Which Norwegian companies had a net income of more than 1,000,000,000 (one billion) Norwegian kroner in 2008, but no more than 4 employees*?
- *Which are the eight oldest accommodation establishments (hotels or other) in Tromsø*?
- *Which companies are settled in Brønnøy, street Industriveien 30*?
- *Which were the seven manufacturing companies of dairy and ice-cream products with the highest net income in 2010*?
- *Which wireless telecommunications companies settled in Oslo have above 40 employees*?
- *Which companies does “Svein Rennemo” lead*?

They had 45 minutes to carry out the search tasks, although all participants were able to finish before the deadline; in fact, the average time for a search task was 8 minutes and 20 seconds (including the time employed filling the search form)–note that participants did not receive any training of PepeSearch.

We used the *F*-measure to assess the retrieval performance, a popular single-valued metric for interactive evaluations [24]. The *F*-measure is calculated as the harmonic mean of recall and precision, ranging from 0 (worst case) to 1 (perfect response). Remarkably, the mean *F*-measure was 0.82 with a standard deviation of 0.17. Participants were also asked to fill a usability questionnaire for assessing subjective reactions to interactive interfaces. We then computed the System Usability Scale (SUS) [25] score, obtaining 75.3 points with a standard deviation of 18.1.

To put our results into context, we have reviewed the literature in order to find user studies on semantic search that could serve as a baseline comparison for PepeSearch. [20] is a comprehensive semantic search usability study with five different user interfaces (2 natural language, 2 graph-based and 1 form-based interfaces). The obtained SUS scores ranged from 32.5 to 63.8 (41.3 in the case of form-based interfaces). Since these SUS scores are not particularly high, we set a goal of 67.0 that is the mean value for web user interfaces found in an extensive study on the use of the SUS questionnaire [26]. We then employed a one-sample *t*-test to assess whether this goal had been accomplished. The observed difference of 8.3 SUS points is 1.8 standard errors from the benchmark, while the obtained *p*-value is 0.048, which is a statistically significant result.

Concerning retrieval performance, we could not use the figures from [20] because the employed dataset was much smaller and the search tasks less complex than in our user study. Instead, we employed as a basis the results from the first and second editions of a public evaluation challenge for question answering systems over linked data (QALD) [27]. Since the focus relies on evaluating natural language processors that automatically translates textual questions into SPARQL queries, users were not involved in the QALD challenges. However, the complexity of the questions are comparable to the ones employed in our case study, e.g. “Give me all live albums by Michael Jackson”, the size of the datasets is comparable, e.g. 15M triples for the MusicBrainz dataset, and the F-measure is also employed for measuring retrieval performance, ranging from 0.38 to 0.71 for the best system with the MusicBrainz dataset in QALD-1. We set this maximum value as a comparison goal in our study, obtaining a difference of 0.11 with a *t* of 2.56 and a *p*-value of 0.011, thus indicating a statistically significant difference.

All in all, this analysis suggests that the usability-expressivity tradeoff of PepeSearch is well balanced, given the high scores for retrieval performance and usability that significantly surpasses the goals we set. Since the comparison with other approaches is mediated through benchmark scores, we aim to conduct further user studies with PepeSearch and other representatives of semantic search interfaces to confirm these results.

### Other settings

Since PepeSearch is designed to be portable, we have tested this tool in other settings. The Semantic Web Dog Food Corpus (http://data.semanticweb.org/) provides information about the main conferences and workshops in the Semantic Web research field. Overall, this dataset is of medium size (about 600K triples) and of moderate complexity (118 classes, 48 object properties and 72 datatype properties). Though the endpoint is not very responsive, it was easy to query and browse this triplestore with PepeSearch, e.g. to find the papers of a researcher in a specific conference.

We also configured PepeSearch to query the Linked Open NPD FactPages [28] dataset (http://sws.ifi.uio.no/project/npd-v2/), which is an RDF representation of a wide range of public information about petroleum related activities on the Norwegian Continental Shelf. This is a large dataset (more than 7M triples) with a rather complex schema about oil fields, involved companies, wells and wellbores—totaling 173 classes, 99 object properties and 349 datatype properties. As in the other cases, no code changes were required to set up PepeSearch. We were also able to query this triplestore, although some domain knowledge is required to understand the FactPages schema.

## Discussion

Given the difficulties that mainstream users have for searching and exploring semantic datasets, we aimed to propose an adequate user interface for their needs. Our preliminary results with PepeSearch show that mainstream users were able to solve non-trivial search challenges with PepeSearch without previous training and that the usability-expressivity tradeoff seems adequate.

The majority of form-based interfaces are still very complicated for lay users, as criticized in [18]. For instance, Explorator [16] shows the subject, property and object constructs of RDF at the user interface, while tFacet [17] employs a hierarchical structure of facets that becomes easily impractical when traversing many classes.

Regrettably, there is a lack of empirical evidence of the effectiveness of query interfaces for the Semantic Web [29]. Rhizomer [18] is one of the scarce proposals that include a user evaluation aimed to test the validity of facet pivoting—this technique allows the formulation of queries with more than one concept (see Section 2). However, they report usability problems as users found problems identifying the pivoting button and making sense of it. In comparison, the interface design of PepeSearch offers a clear separation of classes and additional expressivity, since an arbitrary number of constraints can be set for each query concept.

While dynamic term suggestion is a very popular technique in search interfaces [21], they are not so common in semantic search systems. Participants in our user study profusely employed suggested terms for setting literal string values. As a result, dynamic term suggestion avoided spelling errors and contributed to reduce the number of zero results.

Future work for PepeSearch includes further comparisons with other approaches. In addition, we plan to leverage this tool by supporting new result visualizations and to increase query expressivity without compromising usability. We are also working on supporting basic federation through the use of the SERVICE operator of SPARQL 1.1.

## Supporting Information

S1 File Evaluation data. (XLSX)
